# Identification of nine signature proteins involved in periodontitis by integrated analysis of TMT proteomics and transcriptomics

**DOI:** 10.3389/fimmu.2022.963123

**Published:** 2022-08-09

**Authors:** Wei Liu, Wei Qiu, Zhendong Huang, Kaiying Zhang, Keke Wu, Ke Deng, Yuanting Chen, Ruiming Guo, Buling Wu, Ting Chen, Fuchun Fang

**Affiliations:** ^1^ Shenzhen Stomatology Hospital (Pingshan), Southern Medical University, Shenzhen, China; ^2^ Department of Stomatology, Nanfang Hospital, Southern Medical University, Guangzhou, China; ^3^ Department of Histology and Embryology, School of Basic Medical Sciences, Southern Medical University, Guangzhou, China; ^4^ Shanghai Key Laboratory of Stomatology, Department of Oral Implantology, Shanghai Ninth People Hospital, National Center of Stomatology, National Clinical Research Center of Oral Diseases, School of Medicine, College of Stomatology, Shanghai Jiao Tong University, Shanghai, China

**Keywords:** periodontitis, TMT proteomics, artificial neural network, transcriptomics, inflammation and immune response, signature proteins

## Abstract

Recently, there are many researches on signature molecules of periodontitis derived from different periodontal tissues to determine the disease occurrence and development, and deepen the understanding of this complex disease. Among them, a variety of omics techniques have been utilized to analyze periodontitis pathology and progression. However, few accurate signature molecules are known and available. Herein, we aimed to screened and identified signature molecules suitable for distinguishing periodontitis patients using machine learning models by integrated analysis of TMT proteomics and transcriptomics with the purpose of finding novel prediction or diagnosis targets. Differential protein profiles, functional enrichment analysis, and protein–protein interaction network analysis were conducted based on TMT proteomics of 15 gingival tissues from healthy and periodontitis patients. DEPs correlating with periodontitis were screened using LASSO regression. We constructed a new diagnostic model using an artificial neural network (ANN) and verified its efficacy based on periodontitis transcriptomics datasets (GSE10334 and GSE16134). Western blotting validated expression levels of hub DEPs. TMT proteomics revealed 5658 proteins and 115 DEPs, and the 115 DEPs are closely related to inflammation and immune activity. Nine hub DEPs were screened by LASSO, and the ANN model distinguished healthy from periodontitis patients. The model showed satisfactory classification ability for both training (AUC=0.972) and validation (AUC=0.881) cohorts by ROC analysis. Expression levels of the 9 hub DEPs were validated and consistent with TMT proteomics quantitation. Our work reveals that nine hub DEPs in gingival tissues are closely related to the occurrence and progression of periodontitis and are potential signature molecules involved in periodontitis.

## Introduction

Periodontitis, a bacterially induced, complex, inflammatory disease characterized by continuous destruction of the periodontal soft and hard tissues of the oral cavity, is the main cause of tooth loss in adults (1). Periodontitis is caused by multiple factors and involved in many risk factors making it urgent to explore its mechanism. Previous studies suggest that NLRP3 (2), Transglutaminase 2 (TG2) (3) and periodontal biotype (4), were significant predictors involved in periodontitis or oral diseases related to risk factors of oral health. With the development of omics technology, a variety of transcriptomic and proteomic studies have analyzed periodontitis pathology and progression. However, there is still lacking accurate signature molecules for periodontitis based on multi-omics analysis.

Presently, omics analysis of periodontitis mainly focuses on the orchestration of RNA expression profiling using different tissues (such as GCF, saliva, gingiva and serum), including 5 transcriptomes (5–9), 4 miRNA profiles (10–13), 2 lncRNA profiles (14, 15) and 1 circRNA profile (16). Based on different types of RNA profiles, scholars have gradually revealed a variety of signature mRNAs or ncRNAs that are involved in different periodontitis processes, improving understanding of the pathological mechanisms. However, tremendous amounts of RNA-omics studies attempting to identify specific signature molecules involved in periodontitis have inconsistent results.

In recent years, increasing attention has been given to the proteomics of periodontitis due to the actual function of proteins in various diseases. Ngo et al. first used liquid chromatography-mass spectrometry (LC–MS) to determine the protein composition of GCF and identify 66 proteins, of which 43 were newly reported. Their research had been presented the most comprehensive proteomic study of periodontitis up to 2010 (17). Subsequently, scientists have attempted to describe the protein expression profiles of different samples from the systemic or oral cavity in healthy individuals and periodontitis patients, including saliva (18), dental plaque (19), serum (20) and GCF (21). Nevertheless, few peptides and proteins have been identified in such proteomic studies of periodontitis, and the results were inaccurate due to limitations including low sensitivity, poor separation, and resolution in gel-based MS technology. With the advancement of MS technology, the quantitative proteome technique of tandem mass tags (TMTs) has been widely applied in the analysis of differentially expressed proteins in various diseases based on its deep analysis, good reproducibility and high sensitivity (22). Regardless, few related studies have examined the proteomic profile involved in periodontitis using the TMT proteome technique.

Multi-omics studies play a central role in exploring the pathology and progression of periodontitis, prior investigations have almost focused on the single omics analysis associated with periodontitis. Herein, we first carried out differential expression protein profiling of gingival tissue in healthy individuals and periodontitis patients utilizing quantitative TMT proteomics and found inflammatory immunity-related clusters to be most significantly enriched among 115 DEPs. 9 hub DEPs were screened by LASSO analysis based on proteomics-based protein expression levels. Finally, integrating the transcriptomics of periodontitis, an artificial neural network (ANN) model consisting of 9 hub DEP-coding genes was constructed and confirmed to be effective in distinguishing healthy individuals from periodontitis patients. Furthermore, 9 signature proteins participating in periodontitis were validated with western blotting of human gingiva samples. The results showed that the ANN network of 9 signature molecules identified in our study might be involved in periodontitis, and which may provide a new prediction or diagnosis models for periodontitis.

## Materials and methods

### Study population

The study was conducted in the department of stomatology of Nanfang Hospital. It was approved by the Ethics Committee of Nanfang Hospital, Southern Medical University (No. NFEC-2021-031) and in accordance with the Declaration of Helsinki of 1975, as revised in 2000. The full-mouth and site-specific periodontal parameters for each individual included plaque index, bleeding on probing (BOP), pocket probing depth (PPD), and clinical attachment loss (CAL). The periodontitis and periodontal healthy were diagnosed by periodontal and X-ray examination according to the periodontitis case definition of 2017 World Workshop (23). Inclusion criteria of participants included over 18 years of age, systemic health, with Stages II/III/IV periodontitis; and retaining > 20 teeth equally distributed in all quadrants and >6 teeth per quadrant. Exclusion criteria included smoking, the presence of cardiovascular and respiratory diseases, diabetes mellitus, HIV infection, systemic inflammatory conditions or nonplaque induced oral inflammatory conditions, and immunosuppressive chemotherapy, and pregnancy or lactation. Patients with a history of periodontal therapy or taking medication such as antibiotics or anti-inflammatory drugs that could affect their periodontal status for at least 6 months prior to the study were excluded. Patients unable to maintain sufficient oral hygiene were also excluded from the study. Finally, 10 periodontitis patients and 5 periodontal healthy individuals were included. The basic information of the 15 individuals included in the study is shown in **Table 1**.

**Table 1:** The basic information of the 15 individuals included in proteomic analysis.

Sample	Age		Gender	Diagnosis	Smoking	Tooth loss	Treatment stage	Full-mouth			Site-specific		
								Mean PPD(range; mm)	Mean CAL(range; mm)	BOP(%)	Mean PPD(range; mm)	Mean CAL(range; mm)	BOP(+/-)
P	1	48	Female	Periodontitis	No	0	Before initial therapy	5.15 (3–10)	5.24 (3–11)	57.14	8.40 (6–10)	8.50 (6-10)	+
	2	48	Male	Periodontitis	No	0	Before initial therapy	6.95 (3-10)	7.39 (3-10)	75.00	8.50 (7-9)	8.00 (7-9)	+
	3	41	Male	Periodontitis	No	1	Before initial therapy	5.31 (2-9)	5.54 (2-11)	82.14	8.20 (7-10)	8.8 (7-10)	+
	4	55	Female	Periodontitis	No	1	Before initial therapy	6.81 (4-10)	7.72 (4-11)	85.71	9.00 (8-10)	9.33 (9-10)	+
	5	58	Male	Periodontitis	No	6	Before initial therapy	5.35 (3-10)	5.81 (3-11)	59.52	7.75 (6-9)	8.25 (7-9)	+
	6	38	Female	Periodontitis	No	0	Before initial therapy	3.42 (2-5)	3.83 (0-4)	39.88	4.00 (3-5)	3.50 (3-4)	+
	7	44	Male	Periodontitis	No	0	Before initial therapy	3.55 (2-5)	3.75 (0-4)	36.31	4.50 (3-5)	3.60 (3-4)	+
	8	41	Male	Periodontitis	No	0	Before initial therapy	3.67 (3-6)	3.80 (0-4)	26.19	4.80 (4-5)	3.60 (3-4)	+
	9	58	Male	Periodontitis	No	0	Before initial therapy	3.49 (2-6)	3.66 (0-4)	28.57	4.25 (4-5)	3.17 (2-4)	+
	10	35	Female	Periodontitis	No	0	Before initial therapy	3.27 (2-5)	3.41 (0-4)	38.69	4.50 (4-5)	3.75 (3-4)	+
H	1	34	Female	Healthy	No	0	During CLP	2.02 (1-4)	0.89 (0-2)	4.17	2.75 (2-3)	0	–
	2	43	Female	Healthy	No	0	During CLP	2.10 (1-3)	0.30 (0-1)	4.76	2.67 (2-3)	0	–
	3	37	Male	Healthy	No	0	During CLP	2.05 (1-3)	0.48 (0-1)	5.95	2.20 (1-3)	0	–
	4	38	Male	Healthy	No	0	During CLP	2.08 (1-3)	0.71 (0-1)	6.54	2.00 (1-3)	0	–
	5	34	Male	Healthy	No	0	During CLP	2.01 (1-3)	0.58 (0-2)	7.74	2.25 (2-3)	0	–

P, Periodontitis; H, Healthy individuals; CLP, crown lengthening procedure; PPD, periodontal probing depth; CAL, clinical attachment loss; BOP, bleeding on probing.

### Collection of gingival tissue

Gingival tissues were collected from one site of 10 periodontitis patients and 5 periodontal healthy individuals. The detail for 15 sites was listed in **Table 1**. For the patients with periodontitis, gingival tissue specimens were obtained from periodontal inflamed teeth with no prior supra- or sub-gingival instrumentation. BOP was shown in the sites of biopsies. The PPD at these sites was greater than 4 mm, and radiographic evaluation revealed alveolar bone destruction. In resected sites, sufficient attached gingiva was ensured. Gingival tissue in the periodontal healthy group was obtained during a crown lengthening procedure (CLP). No BOP was observed at these sites, and the PPD was 1-3 mm. No alveolar bone resorption was observed. The gingival samples were resected under local anaesthesia: an internal oblique incision was made, and the resected tissue included gingival epithelium, sulcus epithelium, gingival connective tissue and inflammatory granulation tissue. After resection, the gingival tissue was washed with normal saline and stored at -80°C until used for analysis (24).

### Tandem mass tag (TMT) proteomics

After protein was extracted from the gingival sample, the concentration was determined using the bicinchoninic acid (BCA) method. Protein samples were analyzed by 12% SDS–PAGE (sodium sulfate polyacrylamide gel electrophoresis), and the quality of the samples was evaluated in accordance with the requirements of subsequent experiments. Protein samples with qualified quality were treated with reductive alkylation. The same amount of protein was taken from each sample for trypsin hydrolysis. The peptides were labelled with TMT reagent and mixed in equal amounts. The mixed peptides were preseparated using a C18 reverse-phase column. Liquid chromatography coupled with tandem mass spectrometry (LC–MS/MS) analysis was performed. The search library in the Sequest or Mascot module of ProteomeDiscoverer™ Software 2.4 was used for raw data identification and analysis (25). The filter parameter is Peptide false discovery rate (FDR) ≤0.01.

### Identification of differentially expressed proteins

We first compared the data of the periodontitis group (P group) and healthy group (H group), differentially expressed proteins (DEPs) defined as |log2-fold-change (FC) | > 1 and adj. *p* values < 0.05 were identified using the R package “DEqMS” (26). The DESeqMS package is able to estimate different prior variances for proteins quantified by different numbers of PSMs/peptides, which were used to process and analyze proteomic data. A volcano plot and heatmap were used to visualize the differential results using the R packages “ggplot2” and “pheatmap”, respectively.

### Functional enrichment analysis and protein–protein interaction (PPI)

Functional and pathway enrichment analyses of DEPs were conducted using the Metascape online website (https://metascape.org). Significant Gene Ontology (GO) terms and Kyoto Encyclopedia of Genes and Genomes (KEGG) pathways (both *p* value and *q* value were less than 0.05) were represented using the R package “GOplot”. The PPI network was constructed with the online tool Search Tool for Retrieval of Interacting Genes/Proteins (STRING) (https://string-db.org/) and visualized using Cytoscape software 3.9.0. Subsequently, we divided the PPI network of DEPs into subnetworks through a k-means clustering algorithm, and GO enrichment analysis was applied to identify characteristic biological processes for DEPs in sub-networks.

### Least absolute shrinkage selector operator (LASSO) regression analysis

To screen out hub DEPs, least absolute shrinkage selector operator (LASSO) regression analysis was employed with the R package “glmnet.” Cross-validation was performed to screen the optimal tuning parameter (λ). The minimum log(λ) value was determined as the candidate number of variables. Finally, the combination of predictors (hub DEPs) was analyzed by LASSO regression.

### Transcriptome data acquisition and infiltration of immune cells

Transcriptome data from gingival tissue samples were acquired from two publicly available datasets from the Gene Expression Omnibus (GEO) database: 183 periodontitis and 64 healthy samples from the GSE10334 dataset and 241 periodontitis samples and 69 healthy samples from GSE16134. The Affymetrix probe ID from the microarray data was annotated to gene symbols according to the GPL570 platform. Next, we used the normalize BetweenArrays method in the R package “limma” (27) to normalize the gene expression matrix. The “Comabat” method in the R package “Sva” was used to eliminate the batch effect. The resulting pooled dataset contained 424 periodontitis and 133 healthy samples. Subsequently, we performed Principal Component Analysis (PCA) on the gene expression profile between the GSE10334 and GSE16134 datasets after batch correction. Expression of differentially expressed genes (DEGs) was visualized using the R package “pheatmap”.

Next, single-sample gene set enrichment analysis (ssGSEA) was performed to quantify the relative abundance of immune cell types in the periodontitis microenvironment. The relative abundance of each immune cell type was represented by an enrichment score in ssGSEA and normalized to unity distribution from 0 to 1. The R package “ggcor” (https://github.com/xukaili/ggcor) was used to visualize the correlation between hub DEPs and immune cells.

### Artificial neural network (ANN) analysis

We applied GSE10334 as a training dataset to construct an ANN model based on the “neuralnet” R package (28). As a validation dataset, GSE16134 was used to verify the classification efficiency of the model score constructed with gene expression and gene weight. Before training the ANN model, the datasets were filtered and normalized by min-max normalization. Next, the processed training data were input into the neural network model; the number of neurons should be between the input layer size and the output layer size, usually two-thirds of the input size. Finally, hub genes were inputted and hidden layers, and two outputs (healthy and periodontitis) were set. The output of the first hidden layer (input of the last output layer) in the network results was considered as the gene weight. In this model, the sum of the product of the weight scores multiplied by the expression levels of the important genes was used as the disease classification score. The 5-time cross-validation results display the model classification performance using receiver operating characteristic (ROC) curve analysis. The areas under the ROC curves (AUCs) of the five cross-validation results illustrated the diagnostic ability of the model, and the “pROC” R package was used to calculate and draw the AUC classification performance results.

### Western blotting validation

Total protein (20-40 μg) from human gingival tissues (n=4) was separated by 10% SDS–PAGE and then transferred onto a 0.45-μm polyvinylidene difluoride membrane (Millipore) at 250 mA for 2 h on ice. The membrane was blocked with 5% skim milk dissolved in Tris-buffered saline containing 0.05% Tween 20 and probed with primary antibodies against CD38 (cat# sc-374650), CD79A (cat# sc-20064), ADPGK (cat# sc-100751) were purchased from Santa Cruz Biotechnology (Dallas, Texas, USA), OGN (cat# 12755-1-AP), HLA-DPA1 (cat# 16109-1-AP), PLCH1 (cat# 19143-1-AP), GAPDH (cat# 10494-1-AP) were purchased from Proteintech (Wuhan, Hubei, P.R.C), TMED5 (cat# SRP08852), GSTCD (cat# SRP11511) were purchased from Saier Biotechnology (Tianjin, P.R.C) and TBXAS1 (cat# ab157481) was purchased from Abcam (Cambridge, MA, USA) at the indicated dilutions overnight at 4°C. The membrane was then incubated with horseradish peroxidase (HRP)–conjugated secondary antibodies (anti-rabbit, cat# B900210 and anti-mouse, cat#SA00001-1) were purchased from Proteintech (Wuhan, Hubei, P.R.C) and detected by enhanced chemiluminescence reagents (Biosharp Life Sciences) using an image analyser. Levels of target proteins were normalized to GAPDH, which served as a reference control. The intensity of the protein bands was analyzed with ImageJ software, and the values are expressed as the mean ± standard deviation (SD).

### Statistical analyses

Unpaired Student’s t test was used to compare two groups with distributed variables. Band intensity in western blot images was quantified with ImageJ software, and values are expressed as the mean ± SD. All statistical analyses were performed using R software (Version 4.1.2, https://www.r-project.org/). Significance was determined at *p <*0.05.

## Results

### Summary of TMT proteomics analysis

**Figure 1:**
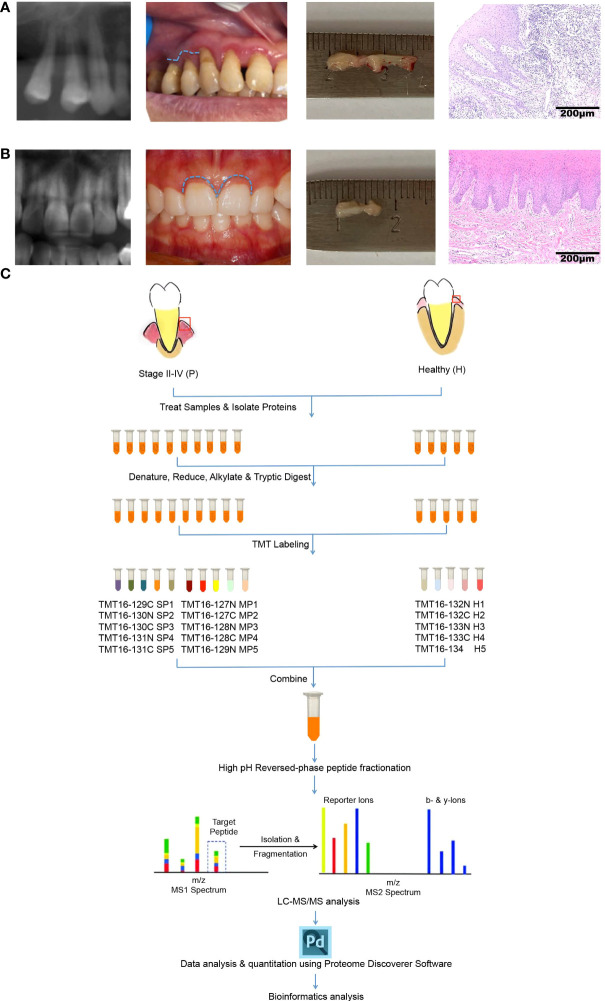
Characteristics of gingival tissues from healthy individuals and periodontitis patients and a schematic overview of the quantitative TMT proteomics workflow. **(A, B)** Collection and H&E staining of gingival tissues from healthy individuals **(A)** and periodontitis patients **(B)**. **(C)** Flow diagram of TMT-based quantitative proteomics.

The sampling operation and Hematoxylin-Eosin (H&E) staining of gingival tissues from healthy or periodontitis populations are shown in **Figures 1A** **, B**. The workflow of TMT-label quantitative proteomics analyses of 15 gingival tissues from healthy or periodontitis individuals is diagrammed in **Figure 1C**. By using high-throughput technology, we identified 45,447 unique peptides, with a FDR < 1%, covering 5658 protein groups. The mass spectrometry proteomics data have been deposited at ProteomeXchange Consortium (http://proteomecentral.proteomexchange.org) *via* the iProX partner repository with the dataset identifier PXD031302 (29).

**Figure 2:**
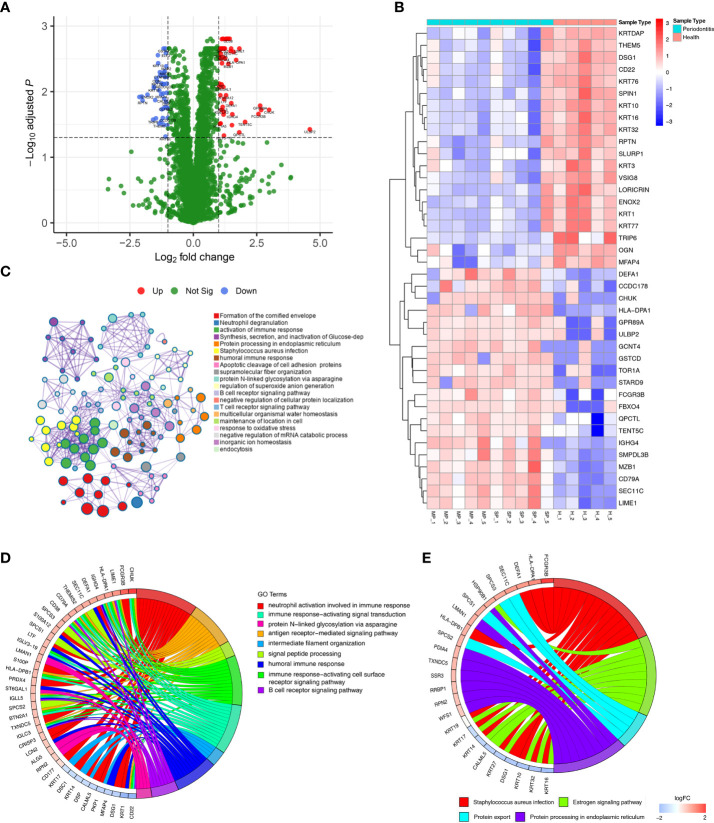
DEPs were identified in gingival tissues from periodontitis versus healthy tissues. **(A)** Volcano plot displaying DEPs in gingival tissues. Green dots indicate down-regulated DEPs, and red dots indicate up-regulated DEPs. **(B)** Heatmap of the top 30 DEPs. Red cells represent up-regulated DEPs, and blue cells represent down-regulated DEPs. **(C)** Metascape enrichment network visualization shows the intra-cluster and inter-cluster similarities of enriched terms for the 115. Nodes of the same color belong to the same cluster. Terms with similarity scores > 0.3 are linked by edges, and nodes in the same condensed network are colored with p values. **(D, E)** Circos plots represent significantly enriched GO terms **(D)** and pathways **(E)** associated with DEPs.

Differential expression analysis showed that 115 proteins were differentially expressed between the healthy group and the periodontitis group, with 66 being up-regulated and 49 down-regulated (shown in **Supplementary Table 1**). These results are visualized by a volcano plot, in which red dots indicate proteins that were significantly up-regulated, blue dots indicate proteins that were significantly down-regulated, and green dots indicate proteins with no differential expression (**Figure 2A**). The DEP expression levels of the top 20 up-regulated and top 20 down-regulated proteins are illustrated by the heatmap in **Figure 2B**, where red represents up-regulated proteins and blue down-regulated proteins.

### Functional characterization of DEPs

GO and KEGG enrichment analyses of all 115 DEPs were performed to explore their biological functions **(** **Figure 2C** **)**. We found terms related to inflammation and immune function to be the most abundant, such as neutrophil degranulation, activation of immune response, human immune response, and B/T-cell receptor signalling pathway.

DEPs were enriched in 9 significant GO terms, as illustrated in **Figure 2D**, with a focus on inflammation-immune related processes, including neutrophil activation involved in immune response, immune response-activating signal transduction, protein N−linked glycosylation *via* asparagine, antigen receptor-mediated signalling pathway, intermediate filament organization, signal peptide processing, humoral immune response, immune response-activating cell surface receptor signalling pathway and B-cell receptor signalling pathway. These processes have been defined as being closely related to periodontitis.

The significant biological pathways based on KEGG enrichment analysis are visualized in **Figure 2E**. The 115 DEPs revealed 4 significantly enriched pathways (*p* value<0.05) in which *Staphylococcus aureus* infection signalling pathway, including FCGR3B and HLA-DPA1, exhibited a significant difference in gingival tissues from healthy and periodontitis groups.

### Protein–protein interaction (PPI) network analysis of DEPs

**Figure 3:**
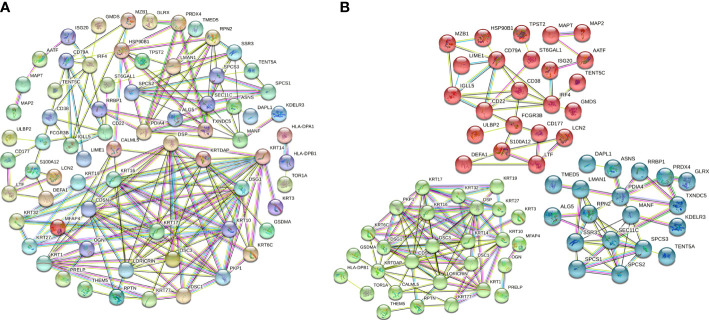
PPI network and subnetwork of DEPs. **(A)** PPI network of 115 DEPs constructed using STRING. **(B)** Functional sub-network analysis of the PPI network.

To further evaluate interactions between the DEPs identified, we used the STRING website to construct a PPI network (**Figure 3A**), which consisted of 174 edges and 112 nodes, with an average node degree of 3.11. We divided the PPI network of DEPs into 3 subnetworks using k-means clustering functional module to understand the core biological functions of the network (**Figure 3B**), and GO enrichment analysis was used to identify characteristic biological processes for DEPs in subnetworks (**Supplementary Table 2**). Among them, 45 DEPs were clustered into the red PPI subnetwork I, which was enriched in immunomodulation-related biological processes, including B-cell receptor signalling pathway, antigen receptor-mediated signalling pathway, and innate immune response. 43 DEPs were clustered into the green PPI subnetwork II, with enrichment in the biological process of protein cornification. The remaining 24 DEPs were clustered into the blue PPI subnetwork III, which was enriched in the biological process of protein processing and transport.

### Identification of 9 hub proteins by LASSO

**Figure 4:**
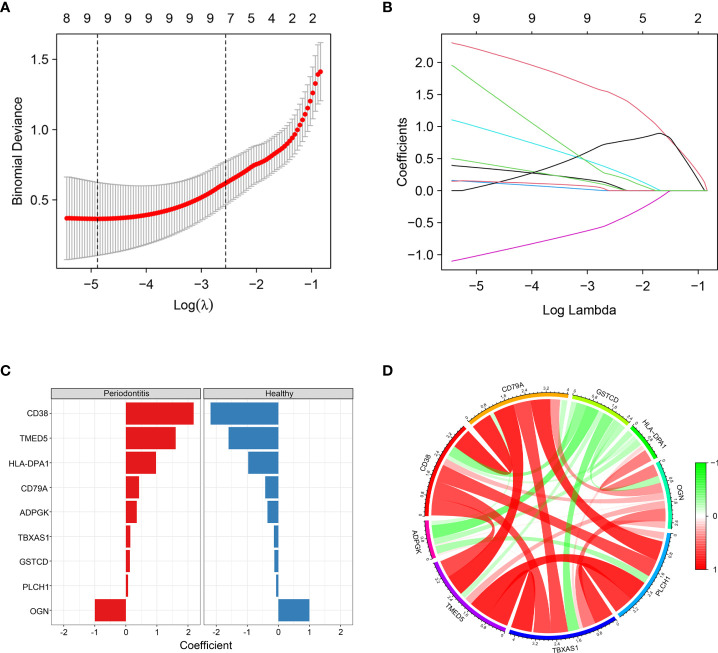
Screening of hub DEPs using LASSO regression analysis. **(A)** Coefficients of nine DEPs were selected by the lambda with the minimum binomial deviance marked by the black dashed line (ln(lambda) = -4.88). **(B)** The LASSO binomial model fitting process. Each curve represents a variable. **(C)** Coefficient values for each of the nine selected proteins from LASSO regression. A positive coefficient for a protein signature within its class indicates that elevated expression of this protein increases the probability of a specimen belonging to its tissue type. **(D)** Circos plot shows the correlations between the nine hub DEPs according to the protein expression levels. Green connecting lines represent negative correlations, and red lines represent positive correlations.

To further identify hub DEPs, we performed LASSO regression analysis based on the 115 DEPs between the healthy and periodontitis groups. As depicted in **Figure 4A**, we identified a binomial classifier for all samples, including the healthy and periodontitis samples. This classifier was based on the expression signatures of 9 DEPs (**Figure 4B**), and DEPs with non-zero coefficients for each class were found to be almost mutually exclusive (**Figure 4C**). Finally, 9 hub DEPs (CD38, TMED5, HLA-DPA1, CD79A, ADPGK, TBXAS1, GSTCD, PLCH1, and OGN) were selected among the 115 DEPs. The coefficient values of 9 hub DEPs for LASSO analysis are shown in **Figure 4C**. In addition, interaction among 9 hub DEPs according to the protein expression levels is presented in **Figure 4D**. We found that GSTCD displayed a strongly negative correlation with the remaining proteins, particularly the strongest negative correlation with ADPGK. ADPGK also showed a negative correlation with PLCH1, HLA-DPA1 and CD79A. Conversely, CD79A, CD38, TMED5, TBXAS1, PLCH1, and OGN showed a positive correlation.

### Expression profiles of 9 hub DEPs in GEO transcriptome datasets

**Figure 5:**
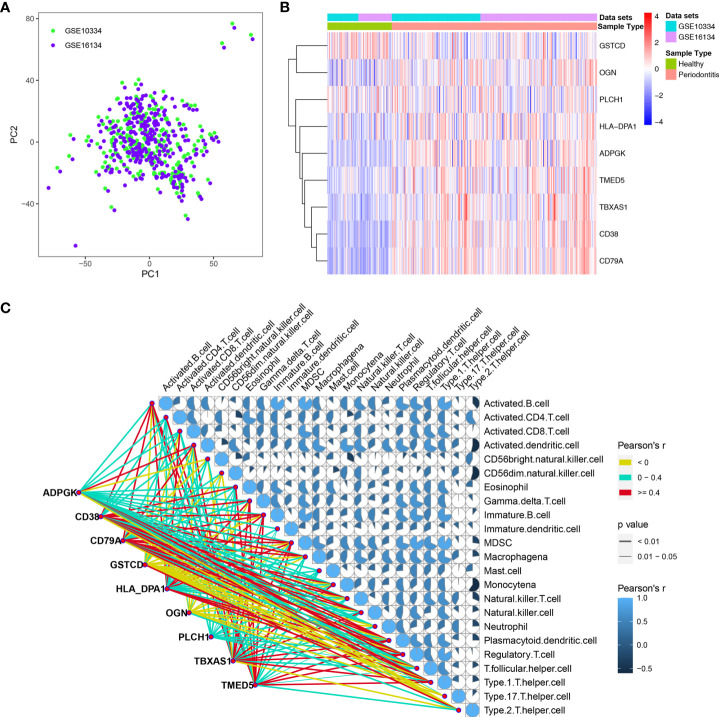
Dataset preprocessing and differential expression analysis of 9 hub DEGs. **(A)** The PCA plot displays removal batch effect between GSE10334 and GSE16134 cohorts. **(B)** Expression profile of the 9 hub DEGs in the “pooled” dataset. **(C)** Expression of 9 hub DEGs correlated with the infiltration levels of various immune cells in periodontitis. The size and color of the pie chart were related to the correlation for the interaction of immune cells. The line color is related to the degree of correlation, and line size represents the p value.

To explore changes in the genes corresponding to these nine hub DEPs, we obtained transcription data from the GEO database, and two independent datasets, GSE10334 and GSE16134, were downloaded for analysis. We normalized and combined the gene expression profiles obtained from the GSE10334 and GSE16134 datasets and then performed batch correction. The resulting pooled dataset contained 424 periodontitis and 133 healthy samples (**Supplementary Table 3**). PCA demonstrated homogeneity in the expression profile between GSE10334 and GSE16134 after removing the batch effect (**Figure 5A**). Compared with the healthy group, the genes encoding the 9 hub DEPs, as DEGs, were significantly differentially expressed in gingival tissues from the periodontitis group in both datasets (**Figure 5B**). Detailed expression profile information of the 9 hub DEGs in the GSE10334 and GSE16134 datasets can be found in **Supplementary Table 4**.

We further determined the level of immune cell infiltration in each sample from the GEO cohort using the ssGSEA method based on transcriptomic data (**Supplementary Table 5**). Next, we calculated correlations between the 9 DEGs and the 23 types of infiltrating immune cells (**Figure 5C**).

### ANN development and verification

**Figure 6:**
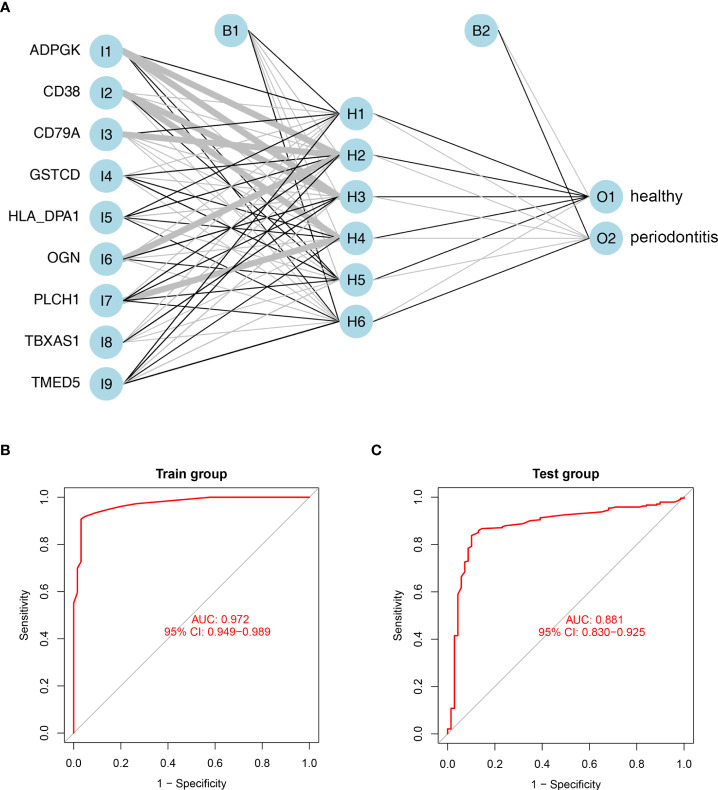
Establishment and validation of artificial neural networks. **(A)** Results of neural network visualization based on the expression of nine hub genes. Linewidths of connectors represent the weights: the wider the line is, the heavier the weight is. O1: healthy group; O2: periodontitis group. **(B, C)** ROC curves for the 9 hub DEGs in the training cohort **(B)** and validation cohort **(C)** by the five-time cross-validation model. AUC, area under the curve.

To construct artificial neural networks of the 9 hub DEPs, the GSE10334 and GSE16134 datasets were used as the training and validation cohorts, respectively. The network architecture is schematized in **Figure 6A** utilizing the training cohort. The colors and linewidths in the figure typify the connections, the weights, and the groups of layers. There were totally 6 hidden layers. Subsequently, we performed ROC analysis of the 9 hub DEGs to predict their sensitivity for periodontitis in the training cohort and the validation cohort respectively. The results of ROC curves showed that the model had relatively high accuracy for the training cohort, with an area under the curve (AUC) of 0.972 (**Figure 6B**). Similarly, ROC curves had an AUC of 0.881 for the validation cohort (**Figure 6C**). These results suggest that this model of 9 hub DEPs is able to effectively distinguish between periodontitis and healthy samples.

### Western blotting validation

**Figure 7:**
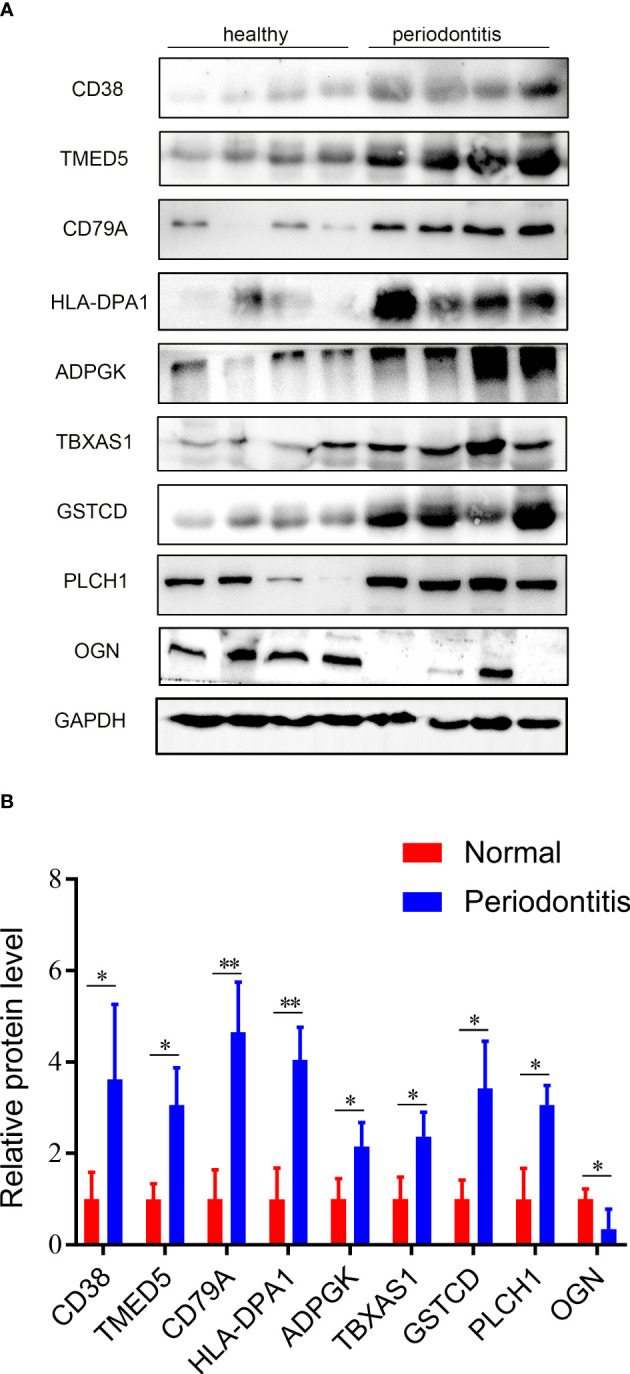
Validation results of western blotting. **(A)** Validation of the 9 hub DEPs by western blotting. GAPDH was used as a loading control. **(B)** Quantitative results of western blotting from Fig 7A. Data are represented as the mean ± SD. *p < 0.05.

Nine hub DEPs, involved in the progression of periodontitis were selected and validated by western blotting using human gingival tissues. Levels of CD38, TMED5, HLA-DPA1, CD79A, ADPGK, TBXAS1, GSTCD and PLCH1 in diseased tissues were significantly increased. However, the protein level of OGN was decreased in periodontal gingival tissues compared with healthy individuals (**Figures 7A** **, B**). These validation results were consistent with the proteomics analysis data.

## Discussion

Previous studies suggest that in the pathogenesis of periodontitis, it begins with the localized inflammation of gingiva. During the occurrence and development of periodontitis, the first pathological change is the inflammation of gingival tissue. Exploring the inflammatory process of gingival tissue is very important to clarify the occurrence and development of periodontitis (30). In this study, we analyzed the TMT-proteomics and transcriptomics of gingiva from periodontitis patients and healthy participants. Finally, we identified nine signature molecules-based on ANN model, which were closely related to the progression of periodontitis.

At present, there are three main comprehensive gene expression/transcriptome profiles for healthy and periodontal gingival tissues, GSE10334, GSE16134, and GSE23586, in the GEO database. Scientists have utilized these datasets to identify specific biological processes involved in periodontitis and analyze significantly DEGs that belong to pathological pathways in the disease (31). Additionally, Kim H et al. investigated differential DNA methylation in gingival tissues of periodontal health, gingivitis, and periodontitis and its association with differential mRNA expression (32), and Richter et al. identified biologically active methylation marks of the oral masticatory mucosa by an epigenome-wide association study (EWAS) (33). These studies have attempted to explore the epigenetic pathological mechanisms associated with periodontitis. In addition to the transcriptomes of periodontitis, researchers have focused on the construction of ncRNA expression profiles (34), especially lncRNA-based ceRNA networks (15).

Compared with in-depth omics studies involving gene expression, there are relatively few studies in this area about proteomics in healthy and periodontal gingival tissues. To date, there are only four reports revealing DEP profiles in human gingival tissue of periodontitis compared with healthy gingiva. Among them, Bertoldi C et al. carried out two-dimensional gel electrophoresis (2-DE) combined with LC–MS/MS to compare the proteomic profiles of inter-proximal pocket tissues and inter-proximal healthy tissues in the same subject at sites where periodontal pathogens were not detectable, and 19 DEPs were identified (35). Monari et al. also used 2-DE with LC–MS/MS to reveal 32 DEPs in periodontal pocket tissue of periodontitis patients compared with the corresponding gingival tissue of periodontal healthy participants (36). Guzeldemir-Akcakanat et al. used comparative proteomic analysis by LC−MS/MS of gingival tissues from chronic periodontitis patients compared with periodontal healthy controls, and found that 319 proteins showed statistically significant expression differences (37). Moreover, Bao et al. used pressure cycling technology (PCT)-assisted label-free quantitative proteomics to explore the DEPs between diseased gingival tissues and healthy control gingival tissues. Finally, 62 up-regulated and 7 down-regulated proteins were identified (38). Although gel-based proteomic strategies mentioned above provide insight into the proteomic landscape of gingival tissues in healthy and periodontitis, they also have serious limitations, including the inability to isolate acidic, basic and hydrophobic (membrane) proteins and a limited number of identified proteins (39).

To overcome these limitations, we first applied advanced quantitative TMT proteomics to characterize the full proteomic profile in 5 healthy and 10 periodontal gingival tissues **(** **Figure 1A** **)**. TMT proteomics has the advantages of accurate quantification, good repeatability, and high sensitivity. Therefore, it is widely utilized in the analysis of DEPs (40). In our study, a total of 45,447 unique peptides covering 5658 proteins were identified in all 15 gingival tissues from healthy and periodontitis patients, far exceeding the number of peptides and proteins identified in the above four proteomics reports based on gel electrophoresis. Next, 115 proteins were differentially expressed in periodontal gingiva compared with healthy gingiva using the R package “DEqMS” method (41). DEqMS is gradually becoming a trend, compared with Student’s t test, ANOVA, Limma and linear mixed-model methods, in the statistical analysis of DEPs in quantitative proteomics, especially TMT proteomics (26). To explore the specific or dynamic protein expression profiles of different degrees of periodontitis, 5 gingival samples from individuals with stage II periodontitis and 5 samples with stage III or IV periodontitis were included in our proteomic study. However, PCA revealed no significant difference in the samples between the SP group and MP group. The differential protein analysis also showed that there was no significant difference in protein expression levels between the two groups **(data not shown)**. These results suggest that the protein expression profiles in gingival tissues with different degrees of periodontitis, at least in our study, were similar.

In periodontitis, inflammatory response occurs as the initiator of a series of events including the host-derived immune response, inflammatory cell adhesion and migration, cell death, proliferation and differentiation (42). According to functional and pathway enrichment analysis, we found the DEPs to be significantly enriched in inflammation- and immunity-related GO terms or pathways, especially neutrophil activation involved in the immune response and *Staphylococcus aureus* infection signalling pathway. These processes have been proven to be closely related to the host local inflammation caused by microbial infection (43), which similarly occurs in the pathology of periodontitis. LASSO regression analysis has usually been employed to screen signature molecules or disease biomarkers in various reports (44). In the present study, we used LASSO analysis to narrow down the 115 DEPs and constructed a binomial classifier on all samples, including healthy and periodontitis samples, based on the expression signatures of 9 hub DEPs. Correlation analysis indicated that some degree of positive or negative correlation existed between the 9 hub DEPs.

CD38 is a non-lineage-restricted, type II transmembrane glycoprotein that synthesizes and hydrolyses cyclic adenosine 5’-diphosphate-ribose, an intracellular calcium ion-mobilizing messenger. Beikler et al. reported that the CD38 gene expression level in gingiva from patients with chronic periodontitis following nonsurgical periodontal therapy was significantly increased compared to that in healthy controls (45). Golijanin et al. found that the number of CD38-labelled plasma cells in gingival biopsy samples from parodontopathy-affected patients was significantly less than that in healthy populations (46). These studies suggest that the gene level of CD38, especially the dynamic changes in CD38-related plasma cells, is closely related to periodontitis. Besides, both gene expression level and protein expression level of CD38 were up-regulated in periodontitis, and this consistent expression trend also suggested it as a representative signature of periodontitis. In summary, we also found that CD38 had the highest correlation with periodontitis among the 9 hub DEPs, which was used as the main variable of the disease classifier.

GSTCD (glutathione S-transferase, C-terminal domain containing) was the most up-regulated protein (log2 fold change: 5.86) among the 9 hub DEPs. The pathway related to GSTCD is Metapathway biotransformation. GO annotations related to this gene include methyltransferase activity and rRNA methyltransferase activity. GSTCD is closely related to lung function (47), though there has beenalmost no reports on the relationship between GSTCD and periodontitis to date. In our study, gene expression of GSTCD in gingiva from periodontitis individuals was significantly increased in the GSE10334 (logFC=5.58) and GSE16134 (logFC=5.57) datasets compared with healthy populations, which is consistent with our TMT proteomics results. These results suggest that GSTCD may be closely related to periodontitis, and further in-depth exploration is needed.

In addition to the above 2 hub DEPs, there is no direct evidence from previous studies for the remaining hub DPEs (TMED5, HLA-DPA1, CD79A, ADPGK, TBXAS1 and PLCH1) correlating closely with periodontitis, OCN had been reported that it is involved in the physiological or pathological process of periodontitis. Therefore, we attempted to further verify the accuracy of the 9 signature proteins involved in the pathogenesis of periodontitis obtained from our gingival tissue proteome using an ANN model (48). ANNs are one of the current tools with intelligent pattern recognition ability, and their application in the classification and diagnosis of infectious diseases, tumors, hypertension and related diseases (49–51), but not periodontitis, has been reported. Considering that the size of our periodontitis proteomics sample was only 15 and the proteomics sample size was also small in previous reports, we were unable to establish an effective ANN model to classify periodontitis utilizing existing proteomic data.

To address this limitation, we utilized transcriptome data (GSE10334 and GSE16134) in gingival tissues from the GEO database to validate the efficiency of the coding genes for the 9 hub DEP classifying periodontitis. Notably, compared with the healthy group, the genes encoding the 9 hub DEPs were significantly differentially expressed in gingival tissues from the periodontitis group in both the GSE10334 and GSE16134 transcriptome profiles, suggesting the potential correlation of 9 signature molecules with periodontitis. In this study, an ANN model consisting of 9 hub DEP-encoding genes obtained by LASSO analysis was constructed for the first time to efficiently distinguish periodontitis and healthy populations.

Isola et al. [2]. analyzed the association between serum and salivary NLRP3 concentrations in patients with periodontitis and type-II diabetes mellitus through a clinical trial, and found that NLRP3 had demonstrated a promising biomarker of disease risk in patients with periodontitis and type-II diabetes mellitus. Matarese et al. demonstrate that increased TG2 expression in HPDL cells from periodontitis patients could be associated with high levels of pro-inflammatory markers promoting the bone remodeling and resorption [3]. Wu et al. reported salivary biomarkers for diagnosing periodontitis using the Spearman rank correlation coefficient with logistic regression and found that the combination of IL-1β, IL-1Rα, and MMP-9 exhibited the highest AUC (0.853), with high sensitivity and specificity for diagnosing periodontitis (52). In our ANN model, the 9 hub DEGs exhibited AUCs of 0.972 and 0.881 (higher than those reported by Wu et al.) for the training and validation cohorts, respectively, and showed that this ANN model had satisfactory classification capacity. Finally, we verified the expression of 9 hub DEPs in gingiva using western blotting. Overall, how these signature molecules affect the pathogenesis of periodontitis needs to be further explored. In particular, classical biological experiments and clinical trials are required to explore the role and underlying mechanism of those promising biomarkers above involved in periodontitis.

In summary, we first determined the protein profiles of gingival tissue in periodontitis and healthy individuals utilizing quantitative TMT proteomics and discovered 9 hub proteins involved in periodontitis by integrated proteomics and transcriptomics analysis using LASSO. The ANN model based 9 signature molecules showed satisfactory classification ability for distinguish healthy from periodontitis patients. This study provides novel insight into potential signature molecules in gingival tissue to predict or diagnosis periodontitis.

## Data availability statement

The datasets presented in this study can be found in online repositories. The names of the repository/repositories and accession number(s) can be found in the article/**Supplementary Material**.

## Ethics statement

The studies involving human participants were reviewed and approved by Ethics Committee of Nanfang Hospital, Southern Medical University (No. NFEC-2021-031). The patients/participants provided their written informed consent to participate in this study.

## Author contributions

WL, WQ, BW, TC, and FF contributed to the conception and design of the research. WL, WQ, and FF contributed to the writing and drafting of the manuscript. ZH, KZ, YC, RG, and KW contributed to draw the figure, table and analysis the data. KD, BW, and TC contributed to the critical revision of the manuscript for important intellectual content. All the authors have approved the final version of the manuscript to be published and agree to be accountable for all aspects of the work.

## Funding

This research was supported by grants from National Natural Science Foundation of China [Grant Nos. 82101024 (WQ)]; Natural Science Foundation of Guangdong Province [Grant Nos. 2020A1515011455 (FF) and 2020A1515110027 (WQ)].

## Acknowledgments

All claims expressed in this article are solely those of the authors and do not necessarily represent those of their affiliated organizations, or those of the publisher, the editors and the reviewers. Any product that may be evaluated in this article, or claim that may be made by its manufacturer, is not guaranteed or endorsed by the publisher.

## Conflict of interest

The authors declare that the research was conducted in the absence of any commercial or financial relationships that could be construed as a potential conflict of interest.

## Publisher’s note

All claims expressed in this article are solely those of the authors and do not necessarily represent those of their affiliated organizations, or those of the publisher, the editors and the reviewers. Any product that may be evaluated in this article, or claim that may be made by its manufacturer, is not guaranteed or endorsed by the publisher.

## Supplementary material

Supplementary Table 1 Proteomic profiles of 115 DEPs identified by DEqMS analysis.

Supplementary Table 2 GO terms for DEPs in PPI sub-networks.

Supplementary Table 3 The resulting pooled dataset contained 424 periodontitis and 133 healthy samples obtained from the GSE10334 and GSE16134 datasets.

Supplementary Table 4 Detailed expression information of the 9 hub DEGs in the GSE10334 and GSE16134 datasets.

Supplementary Table 5 The level of immune cell infiltration in each sample from the GEO cohort by the ssGSEA method based on transcriptomic data.
